# Addressing Alcohol Use Disorder in Alcohol-Associated Liver Disease

**DOI:** 10.14309/ctg.0000000000001038

**Published:** 2026-04-17

**Authors:** Sheel Patel, Nimish Thakra, Akash Shukla, Manisha Verma, Ashwani K. Singal

**Affiliations:** 1 Department of Medicine, University of Louisville, Louisville, Kentucky, USA;; 2 Division of Digestive Diseases and Nutrition, University of Kentucky, Lexington, Kentucky, USA;; 3 Sir HN Reliance Foundation Hospital, Raja Ram Mohan Roy Marg, Girgaon, Mumbai, India;; 4 Department of Medicine, Thomas Jefferson University, Philadelphia, Pennsylvania, USA;; 5 Division of Gastroenterology Hepatology and Nutrition, University of Louisville, Louisville, Kentucky, USA;; 6 Trager Transplant Center at Jewish Hospital, University of Louisville, Louisville, Kentucky, USA;; 7 Robley Rex VA Medical Center, Louisville, Kentucky, USA.

**Keywords:** AUD, brief intervention, abstinence, ALD, AH

## Abstract

Alcohol use disorder (AUD) is a prevalent public health challenge currently. Alcohol-associated liver disease, the commonest end-organ damage, is increasing at an alarming rate. It is currently the leading cause worldwide of hospitalization, liver transplantation, and death from cirrhosis of the liver. Furthermore, AUD in patients with liver disease remains untreated, which consequently accelerates liver injury and worsens patient-related outcomes and healthcare burden. It is therefore very critical for all clinicians and healthcare providers to screen for and address AUD at any healthcare encounter. In this review, we provide strategies and tools for clinicians to use in the clinic to effectively provide holistic, comprehensive treatment for AUD and liver disease in patients with alcohol-associated liver disease.

## INTRODUCTION

In 2019, as per the World Health Organization report, alcohol use was reported in 44% of individuals worldwide and 60% in the United States. (1,2). The corresponding figures for alcohol use disorder (AUD), an impaired ability to stop or control alcohol use, despite adverse social, occupational, or health consequences, are 7% and 10.2%, respectively. Although chronic harmful alcohol use causes multisystemic negative effects, alcohol-associated liver disease (ALD) is the most common end-organ damage from alcohol, with 75%–80% patients with ALD having moderate to severe AUD (≥4 of Diagnostic Statistical Manual-5 [DSM-5] criteria), (3). ALD is estimated to cause approximately 3 million deaths annually, representing 5.3% of all global deaths and 13.5% of deaths among adults aged 20–39 (4,5). ALD is currently the leading indication for liver transplantation in the United States, accounting for more than 40% of all transplant listings (6,7). Furthermore, patients with ALD often present at an advanced stage, with missed opportunities at healthcare encounters to recognize risk for and presence of ALD at an early stage (8). A new subgroup, metabolic dysfunction and alcohol-related liver disease, reflects the overlap between metabolic risk factors and alcohol use. Recognition of this new phenotype is important because patients with metabolic risk factors may still have clinically significant alcohol exposure leading to liver injury, underscoring the need for careful alcohol use screening.

Despite the benefits of AUD treatment to reduce the development of ALD and improve liver-related outcomes in those with any spectrum of ALD (9–11), AUD treatment remains underutilized. The treatment gap for AUD is approximately 90%, significantly higher compared with other psychiatric conditions (12). In patients with ALD, fewer than 1 in 5 receive any intervention, and under 2% are prescribed pharmacotherapy for AUD (13). Patient-level barriers include poor understanding of AUD and perceived social stigma, reducing willingness to seek care (14). The clinician-level barrier is a lack of formal training in addressing and managing AUD. For example, in a survey of 408 hepatology/gastroenterology providers, 71% never prescribed AUD pharmacotherapy, 77% reported a lack of training, and more than 90% affirmed for AUD training (15). System-level barriers are restrictive insurance coverage, inadequate clinic time and care coordination, and treatment cost.

Despite the United States Preventive Services Task Force recommending screening of adults for alcohol use (16), there is no formal existing tool built into electronic medical records for primary care. Clearly, clinicians require a practical and structured approach to screen for alcohol use at a healthcare encounter and screen for liver disease in those with harmful alcohol use. This *Clinical Toolbox* provides a pragmatic framework for clinicians to provide comprehensive, holistic care for the dual pathology of AUD and liver disease in patients with ALD.

ALD is currently the leading cause for cirrhosis in most parts of the world, accounting for more than half of cirrhosis-related hospitalizations and 40%–50% of all liver transplants in the United States, accounting for more than 40% of all transplant listings.

Ongoing alcohol use is the most important determinant of long-term outcomes in patients with ALD. However, treatment for AUD is used in about 10%–20% of ALD patients with medications for AUD used in only 1%–2% patients.

## SCREENING FOR AUD AND FOR ALD

Compared with other liver diseases, ALD is often diagnosed at an advanced stage when liver decompensation (ascites, variceal bleeding, and hepatic encephalopathy) has developed (17,18). Speculated reasons for this observation are poor specificity of liver biochemistry and ultrasound, with missed diagnoses in 40%–75% (19,20), asymptomatic or nonspecific symptoms in more than 90% with ALD, including 70% with compensated cirrhosis (21). This underscores the importance of early screening for AUD and ALD.

**Figure 1.**
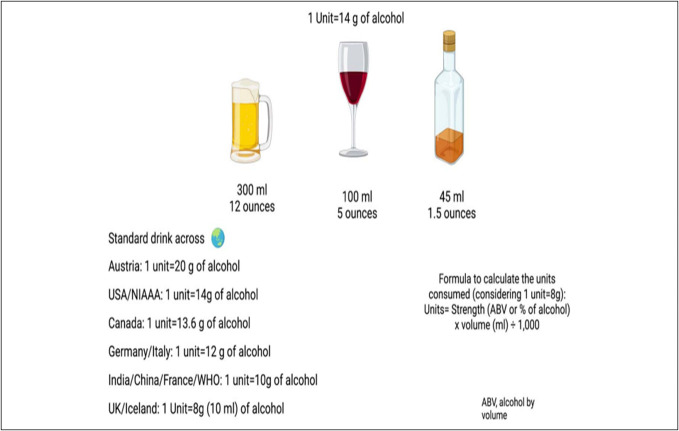
Definition of alcohol-containing drink as defined by NIAAA (Source: Kulkarni and Singal, Clin Liver Dis 2023). NIAAA, National Institute of Alcoholism and Alcoholism Abuse.

**Figure 2.**
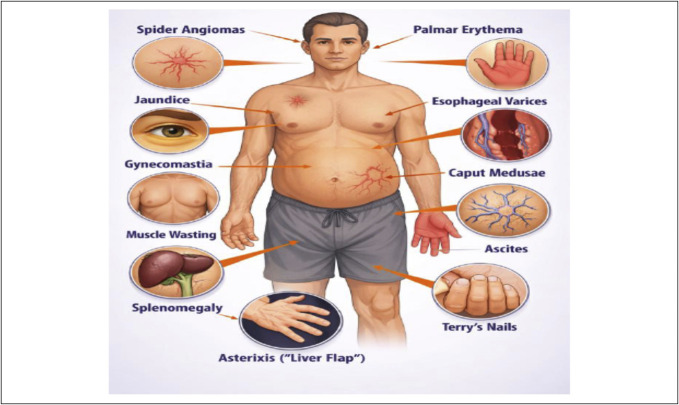
Clinical stigmata of cirrhosis and liver cell failure.

**Figure 3.**
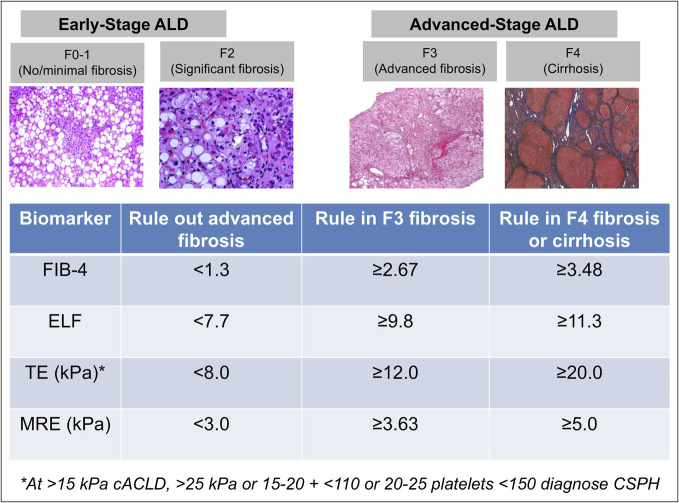
Spectrum of ALD. ALD, alcohol-associated liver disease; FIB-4, fibrosis-4; ELF, enhanced liver fibrosis; MRE, magnetic resonance elastography; TE, transient elastography.

Of several tools available for screening of alcohol use (Table 1), DSM-5 and AUD Identification Test (AUDIT) are most commonly used. AUDIT, a 10-item questionnaire, each self-reported by the patient on a scale of 0–4, is a valid and accurate tool (73–88% sensitivity and 74–100% specificity) for diagnosis and severity of AUD (16). Brief versions such as Alcohol Use Disorders Identification Test-Concise (AUDIT-C) with 3 questions and the Single Alcohol Screening Question are also recommended for use in clinical practice (5). An AUDIT-C cutoff score of >3 and >4 in women and men, respectively, identifies AUD with an accuracy of 73%–97% for women and 82%–100% for men. As these tools report the number of drinks, it is important to convert this to absolute gram of alcohol consumed (Figure 1). DSM-5 tool diagnoses AUD (≥2 of 11 criteria) and grades its severity as its moderate (≥4–5 criteria) and severe (≥6 criteria (22)). DSM-5 does not quantify alcohol use and is based on the physical, social, and professional impact of alcohol use, unlike AUDIT and AUDIT-C, which are more often used for AUD screening. AUD should also be differentiated from dependence, with the development of withdrawal symptoms on abstaining from alcohol. Screening for ALD should be performed in individuals with harmful alcohol use (>30 g/d and >20 g/d in men and women, respectively) or the presence of AUD. Clinical examination for stigmata of liver failure (Figure 2) and decompensation (ascites, hepatic encephalopathy, and variceal bleeding); liver chemistry (aminotransferases such as alanine aminotransferase [ALT] and aspartate aminotransferase [AST] and synthetic function tests such as bilirubin, albumin, and prothrombin time); platelet count; and liver ultrasound are recommended. In individuals with abnormalities on 1 or more of these tests, further assessment of severity is performed using noninvasive tests, such as the fibrosis-4 score (serum-based) and transient elastography using Fibroscan (imaging-based) for liver stiffness measurement (LSM) in kilopascals (kPa) (23). If needed, additional, more accurate serum tests (e.g., enhanced liver fibrosis score) and imaging (e.g., MR elastography) can be used (Table 2). The disease spectrum (Figure 3) extends from early stage (F0-2), advanced fibrosis or cirrhosis (F3-4) with or without significant portal hypertension. A high index of suspicion is needed for alcohol-associated hepatitis (AH) among those with serum total bilirubin>3 mg/dL, with severity stratified to moderate or severe form at a model for end-stage liver disease score cutoff at 21. Those with severe diseases should be assessed for organ failures and acute-on-chronic liver failure.

**Table 1.** Tools to screen for alcohol use disorder

(A) DSM V criteria	
Domain	DSM-5 criteria (≥2 symptoms within 12 mo)
Impaired control	• Alcohol is often taken in larger amounts or over a longer period than intended. • Persistent desire or unsuccessful efforts to cut down or control use. • A great deal of time spent obtaining, using, or recovering from alcohol. • Craving or a strong desire/urge to use alcohol.
Social impairment	• Recurrent alcohol use resulting in failure to fulfil major obligations at work, home, or school. • Continued use despite persistent or recurrent social or interpersonal problems caused or worsened by alcohol. • Important social, occupational, or recreational activities are given up or reduced because of alcohol use.
Risky use	• Recurrent use in physically hazardous situations. • Continued use despite knowledge of a persistent or recurrent physical or psychological problem likely caused or worsened by alcohol.
Pharmacologic criteria	Tolerance: • Need for markedly increased amounts to achieve intoxication/desire effect, or • Markedly diminished effect with continued use of the same amount. Withdrawal: • Characteristic withdrawal syndrome, or • Alcohol (or a related substance such as a benzodiazepine) is taken to relieve or avoid withdrawal symptoms.
Severity specifiers	Mild: 2–3 symptoms Moderate: 4–5 symptoms Severe: 6 or more symptoms

(B) AUDIT and AUDIT-C					
Questions	0	1	2	3	4
**1. How often do you have a drink containing alcohol?**	Never	≤/mo	2–4/mo	2–3/wk	≥4/wk
**2. How many standard drinks do you have on a typical day when you are drinking?**	1–2	3–4	5–6	7–9	≥10
**3. How often do you have 6 or more drinks on 1 occasion?**	Never	</mo	Monthly	Weekly	Daily or almost daily
4. How often during the last year have you found that you were not able to stop drinking once you had started?	Never	</mo	Monthly	Weekly	Daily or almost daily
5. How often during the last year have you failed to do what was normally expected of you because of drinking?	Never	</mo	Monthly	Weekly	Daily or almost daily
6. How often during the last year have you needed a first drink in the morning to get yourself going after a heavy drinking session?	Never	</mo	Monthly	Weekly	Daily or almost daily
7. How often during the last year have you had a feeling of guilt or remorse after drinking?	Never	</mo	Monthly	Weekly	Daily or almost daily
8. How often during the last year have you been unable to remember what happened the night before because of your drinking?	Never	</mo	Monthly	Weekly	Daily or almost daily
9. Have you or someone else been injured as a result of your drinking?	No		Yes, but not in the last year		Yes, during the last year
10. Has a relative, friend, doctor, or other healthcare worker been concerned about your drinking or suggested you cut down?	No		Yes, but not in the last year		Yes, during the last year

AUDIT, Alcohol Use Disorder Identification Test; AUDIT-C, AUDIT-Concise; DSM, Diagnostic Statistical Manual.

AUDIT C (in bold).

**Table 2.** Cutoff scores for NIT in ALD

Test	Cutoff for advanced fibrosis (F3-F4) in ALD	Cutoff of cirrhosis in ALD	Diagnostic performance	Considerations
FIB-4	>2.67 (rule-in); <1.3 (rule-out) (24)	>3.25 (25)	Sensitivity 80%–90%, specificity 60–70% for excluding F3–4 (5)	Accuracy reduced by thrombocytopenia from alcohol use; AST >200 IU/L may overestimate fibrosis (5)
VCTE	≥12.1 kPa (25)	≥15 kPa (21)	Sensitivity 86%, specificity 94% at 15 kPa (21)	Liver stiffness decreases after ∼2 wk of abstinence; elevated AST or bilirubin may falsely elevate values (21)
ELF	≥10.5 (high NPV for rule-out) (26)	≥11.3 (27)	98% negative predictive value at 10.5 (27)	Less affected by active drinking; strong performance in primary care ALD populations (27)
MRE	≥3.5–3.6 kPa (26)	≥4.5 kPa (26)	Sensitivity 0.73, specificity 0.91 for cirrhosis (21)	ALD-specific validation limited; inflammation and elevated GGT may overestimate early fibrosis (26)

ALD, alcohol-associated liver disease; ALT, alanine aminotransferase; AST, aspartate aminotransferase; ELF, enhanced liver fibrosis; FIB-4, fibrosis-4; GGT, gamma-glutamyltransferase; MRE, magnetic resonance elastography; NIT, noninvasive test; NPV, negative predictive value; VCTE, vibration controlled transient elastography.

Screening for AUD and ALD is a cost-effective strategy (28). In a retrospective cohort of 185 veterans with severe AUD screened with Fibroscan, 22 had advanced liver disease, and 41% with LSM  ≥10 kPa were confirmed to have cirrhosis within 6 months and were integrated with specialist hepatology care. Notably, veterans with elevated LSM were more likely to maintain abstinence after 1 year (29). Taken together, screening for alcohol use and for ALD in those at risk identifies ALD at an earlier stage, enhances engagement, creates a window to intervene and improve outcomes to reduce development of ALD, advanced stage in those with early disease; reduce decompensation and irreversible liver injury in those with cirrhosis; and improve survival and quality of life in those with decompensated disease and AH.

Using a brief version of AUD Identification Test (AUDIT-C), screening for alcohol use should be performed by all the providers at every healthcare encounter in clinics, inpatient care, and in emergency department visits.

## MANAGEMENT OF AUD

### Brief intervention

After screening, providers should perform motivational interviewing (MI) and provide brief intervention (BI) to everyone with AUD, and identify those who need treatment referral to addiction medicine specialists. BI, a component of the Screening Brief Intervention and Referral to Treatment pathway, is designed to be used by nonaddiction medicine providers and effectively delivered in person, on the telephone, or on an online platform. It involves personalized feedback by the provider on the patient's alcohol consumption and associated harms (30). Providers should use a nonjudgmental, empathetic, and supportive framework focusing on the patient's intrinsic motivation for behavioral change (Table 3) (30,31).

**Table 3.** Approach for motivational interviewing and brief intervention

Step	Description	Translation to clinical practice
FLAGS approach for brief intervention in alcohol use disorder/harmful alcohol use		
F—Feedback	Give feedback about risks and consequences of drinking	Connect alcohol use to symptoms (eg: sleep issues and laboratory findings) Explain risks of ongoing alcohol use
L—Listen	Listen to the patient's response. Discuss false beliefs regarding the patient's alcohol consumption in relation to guidelines.	Ask open-ended questions Reflect emotions (sounds like alcohol has been your stress-coping mechanism)
A—Advice	Provide personalized, nonjudgmental advice on reducing drinking patterns and associated benefits.	Initiate nonjudgmental discussion (“Let's discuss why cutting down on alcohol would be helpful for you”) Explain the spectrum of health issues associated with alcohol consumption
G—Goals	Discuss safe drinking limits and help the patient to set personal goals for changing drinking patterns.	Define limits (number of drinks/week) Use special occasions as milestones for different goals Explore motivations for change
S—Strategies	Develop a negotiated plan to reach the agreed-upon goal	Offer brief counseling, resources on support groups, or referral to addiction medicine specialists. Plan frequency of follow-up
OARS approach for motivational interviewing		
O—Open-ended question	Encourage the patient to elaborate and explore their own motivations	What concerns do you have about your drinking? How do you think alcohol affects your daily life?
A—Affirmations	Recognize and affirm the patient's strengths and efforts.	It's great that you have been thinking about cutting down. I can see that you are committed to make a change.
R—Reflective Listening	Echo and clarify the patient's sentiments, demonstrating understanding and empathy	Patient: But drinking is a part of my social life. MI: Does socializing always associate with drinking for you?
S—summarizing	Offer a periodic summary of the discussion to ensure mutual understanding	So you are concerned about your drinking habit, but it is also a big part of your social life. You are trying to balance both sides and figure out a healthier way forward, is that right?”

In alcohol-associated liver disease, noninvasive fibrosis assessment must account for the confounding effects of active alcohol use and hepatic inflammation. Aminotransferase-based scores such as FIB-4 are useful for initial risk stratification but may overestimate fibrosis in the setting of thrombocytopenia or elevated AST. Liver stiffness measurements obtained by transient elastography can be transiently elevated during active drinking or acute inflammation and may decrease after short periods of abstinence. Sequential testing strategies, particularly FIB-4 followed by ELF or elastography in indeterminate cases, improve diagnostic accuracy and reduce false-positive results. Noninvasive testing is best performed in stable outpatient settings and should be avoided during alcohol-associated hepatitis.

AST, aspartate aminotransferase; ELF, enhanced liver fibrosis; FIB-4, fibrosis-4; MI, motivational interviewing.

BI and MI are most effective for individuals with mild AUD (AUDIT 8–20). In a study of more than 9,000 individuals with mild AUD in primary care, the number needed to treat was 7 to achieve a reduction of alcohol use within recommended limits in 1 patient (32). A 2018 meta-analysis of 69 studies, 33,642 participants with AUD seen in primary care or emergency care settings showed a reduction of alcohol consumption by 20 (12–28) gm/wk. After BI for 1 year, compared with no or minimal intervention, the effect was most prominent in patients with mild to moderate AUD (33). In a prospective study of 750 patients evaluated in the emergency department, patients with moderate to severe AUD receiving therapist-initiated BI had a steeper decline in alcohol use compared with computer-initiated BI (34). In a meta-analysis of 19 randomized controlled trials, MI combined with psychosocial interventions such as cognitive behavioral therapy achieves the greatest reduction in AUDIT scores among patients with an AUDIT score ≥15 compared with usual care (35).

Using an empathetic and nonjudgmental approach, providers should perform motivational interviewing and provide brief intervention on harms of alcohol use.

### Pharmacotherapy for AUD

Medications for AUD (MAUD) work best when used in combination with behavioral interventions (Table 4). However, clinicians should be aware of MAUD because this is a core component of AUD care, especially for patients with obvious alcohol craving triggers. Combined with BI, MAUD reduces alcohol use, supports abstinence, and improves liver-related outcomes.

**Table 4.** Medications for Alcohol Use Disorder in Alcohol-Associated Liver Disease

Medication	Typical dose	Mechanism	Side effects	Precautions	Use in liver disease
FDA approved medications					
Acamprosate	666 mg PO TID (333 mg TID if CrCl 30–50)	Modulates glutamate signaling	Diarrhea, nausea	Avoid if CrCl <30	Preferred in ALD; not hepatically metabolized
Naltrexone (oral)*	50 mg PO daily	μ-opioid receptor antagonist	Nausea, headache	Avoid acute hepatitis; monitor LFTs	Use cautiously in Child-Pugh A cirrhosis; avoid decompensated disease
Naltrexone (IM)*	380 mg IM monthly	μ-opioid receptor antagonist	Injection site reactions	Cannot rapidly discontinue	Avoid in cirrhosis due to the inability to rapidly discontinue
Disulfiram	250 mg PO daily	Aldehyde dehydrogenase inhibition	Hepatotoxicity, neuropathy	Requires abstinence	Contraindicated in all ALD
Non-FDA-approved medications					
Baclofen	5–10 mg PO TID	GABA-B receptor agonist	Sedation, weakness	Taper slowly	Safest studied agent in decompensated cirrhosis; minimal hepatic metabolism
Gabapentin	300–600 mg PO TID	GABA modulation	Sedation, dizziness	Renal dose adjustment	Generally safe; caution with hepatic encephalopathy
Topiramate	25–300 mg PO daily	GABA enhancement; glutamate inhibition	Cognitive slowing	Slow titration	Limited ALD-specific data; no known hepatotoxicity
Emerging therapies					
Lactobacillus (probiotics)	Variable	Gut microbiome modulation	Minimal	Adjunctive only	Investigational adjunct
Fecal microbiota transplant	Protocol-dependent	Restores gut microbiome; reduces endotoxemia and gut permeability	Infection risk, GI symptoms	Use only in specialized centers; donor screening required	Investigational; early trials suggest potential benefit in severe ALD and alcoholic hepatitis
Metadoxine	500–1,000 mg PO daily (study-dependent)	Enhances ethanol metabolism; antioxidant and anti-inflammatory effects	GI upset, headache	Limited availability; not FDA-approved	Investigational; small studies suggest improvements in liver biochemistry without established outcome benefit
Polyunsaturated fatty acids	Variable	Anti-inflammatory; modulates lipid metabolism	GI intolerance	Supplement quality varies	Investigational adjunct; heterogeneous data suggest potential anti-inflammatory benefit in ALD
Fibroblast growth factor-21 analogs	Trial-based	Regulates alcohol preference and hepatic metabolism	Nausea, GI effects	Research use only	Investigational; early studies suggest modulation of alcohol preference and metabolic pathways relevant to ALD

ALD, alcohol-associated liver disease; GABA, gamma aminobutyric acid; GI, gastro intestinal; LFT, liver function test; TID, three times daily.

A specific MAUD is chosen based on potency, treatment goal, liver disease severity, and patient preference. Of all MAUD, FDA-approved naltrexone and acamprosate are most effective in patients with AUD, although data in patients with ALD are limited. Although there remains a black box warning for use of naltrexone (both oral and depot intramuscular) in patients with advanced ALD, observational data are emerging on its safety in patients with compensated cirrhosis. Acamprosate is not hepatically metabolized and is generally safe in hepatic impairment (dose adjustments or avoidance apply for severe renal dysfunction), making it a preferred option in many patients with liver disease (Center for Substance Abuse Treatment 2009). Disulfiram is contraindicated in any spectrum of ALD because it is fully metabolized by the liver (5).

Of the non-FDA-approved MAUD, gabapentin (endorsed by the American Psychiatric Association) and baclofen (endorsed by the American College of Gastroenterology) are most often used in patients with ALD. Topiramate, endorsed by Vetarans Affairs/Department of Defense guidelines, is an effective therapy, especially for patients with comorbid migraines, epilepsy, or obesity, and those seeking harm reduction rather than abstinence (36). Gabapentin has the advantage of safety because this is not metabolized by the liver and maintains abstinence with a very minimal risk for alcohol withdrawal and sleep/mood disturbances in early recovery (37). However, providers should be aware of its sedation potential, which may limit its use in those with hepatic encephalopathy (38). Of all MAUD, baclofen is studied the most in patients with ALD, including those with decompensated cirrhosis and AH. This MAUD increases abstinence and is also effective in treating alcohol dependence (39). The critical safety concern is life-threatening withdrawal syndrome on abrupt discontinuation; gradual titration is essential, and patients must be explicitly counseled. There are several emerging therapies or repurposed drugs that have the potential for treating AUD. Glucagon receptor-like-1 receptor agonists, Lactobacillus GG, fecal microbiota transplant, metadoxine, polyunsaturated fatty acids, and fibroblast growth factor-21 have a potential for use as MAUD and reducing liver injury (40–45). However, large randomized controlled trials are needed to investigate their benefit and safety in patients with liver disease.

In addition to MAUD, providers should be comfortable in prescribing withdrawal prevention medication in those with a history of or at high risk for withdrawal. A tapered regimen for 5 days of clonazepam, librium, or lorazepam is recommended (46). Caution should be taken to prescribe those with hepatic encephalopathy, given potential worsening, and these patients should ideally be hospitalized for AUD treatment and monitoring withdrawal using the standard Clinical Institute Withdrawal Assessment scale.

In patients with some level of motivation to control ongoing alcohol use, providers should prescribe medications for AUD and community resources to choose programs in their respective communities for additional help and counseling.

## COMMUNITY RESOURCES

In addition to BI/MI and pharmacotherapy, providers should also offer available community and national resources, such as AUD treatment programs, mutual support groups, and online tools. For example, a National Institute of Alcoholism and Alcoholism Abuse alcohol treatment navigator is a comprehensive online tool that helps patients find evidence-based, high-quality AUD treatment programs (inpatient, outpatient, and telehealth) aligned with the patient's residence. Other community resources include Alcoholics Anonymous (AA) (47), Sequential Multiple Assignment Randomized Trial Recovery, LifeRing Women for Sobriety, Secular Organizations for Sobriety, and Secular AA. These groups offer online and in-person patient support groups in accordance with the patient's individual beliefs and preferences. (48) Substance Abuse and Mental Health Services Administration, a national resource, maintains an AUD treatment locator in community health settings (49).

## REFERRAL PATHWAY FOR AUD IN ALD

Patients with moderate to severe AUD (AUDIT >15) and those with significant psychiatric comorbidities should be referred to addiction medicine specialists for AUD treatment. In addition, patients with significant psychiatric comorbidities (bipolar disorder, schizophrenia, severe anxiety or posttraumatic stress disorder, history of suicidal ideations, or attempts), previous failed rehabilitation attempts, and those with substance use other than alcohol except smoking or marijuana will also benefit from addiction specialist medicine care.

**Figure 4.**
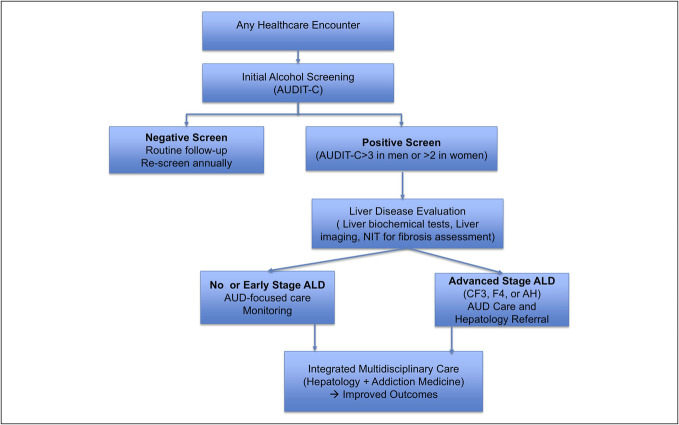
Integrated multidisciplinary care model for managing the dual pathology in patients with ALD. AH, alcohol-associated hepatitis; ALD, alcohol-associated liver disease; AUD, alcohol use disorder; AUDIT-C, Alcohol Use Disorders Identification Test-Concise; NIT, noninvasive test.

Integrated care models incorporating both hepatology and addiction medicine services in the same location provide better results as compared with siloed management of AUD in addiction medicine and of advanced liver disease in hepatology clinics (Figure 4). In a meta-analysis of 20 studies with liver disease, concomitant AUD treatment reduced alcohol use and improved liver-related outcomes such as decompensation, 30-day readmission, and patient mortality (50). Furthermore, in a subgroup of liver transplant recipients for ALD, an integrated care model compared with usual AUD treatment was associated with 52% and 71% lower odds for relapse to alcohol use and for posttransplant patient mortality (50). Most evidence for integrated care models is derived from patients with advanced ALD (decompensated cirrhosis, AH, and transplant candidates). Data on this model are needed in patients who have noncirrhotic or early-stage ALD (50–52).

These models typically involve multidisciplinary teams (hepatology, addiction medicine, psychiatry, and social work) with colocated or coordinated care for patients. Core elements include systematic alcohol use screening (using AUDIT-C/AUDIT), early initiation of pharmacotherapy, structured behavioral interventions, and longitudinal follow-up for monitoring liver disease and alcohol use. Implementation varies across settings, from transplant-centered programs with embedded addiction services to outpatient hepatology clinics using referral-based or hybrid approaches. However, adoption is limited by several factors at the patient level (stigma perception), providers (lack of time and adequate training on AUD), and the administrative or system level (resource constraints and reimbursement). Expanding these models into community settings, supported by telemedicine, may improve access and outcomes across the ALD spectrum (53).

An integrated care model involving hepatology and addiction specialists should be promoted to manage the dual pathology in and provide comprehensive holistic care to patients with ALD especially those with moderate to severe AUD.

### Psychosocial treatment

For effective management of moderate to severe AUD, a combination of MAUD (*see earlier section*) and structured behavioral support by counselors and addiction experts addresses motivation, coping skills, and social reinforcement (51). Meta-analyses and systematic reviews demonstrate that integrated approaches combining psychosocial therapy with medical care yield higher abstinence rates (up to 74%) compared with usual care (48%) (51).

Behavioral therapies, including cognitive behavioral therapy, motivational enhancement therapy, and 12-step facilitation, are most effective when provided as part of a coordinated program alongside medical care (52). These approaches target behavioral change, motivation, and social support and are often delivered in individual, group, or integrated multidisciplinary formats. In particular, motivational enhancement therapy is repeatedly associated with improved outcomes in ALD/AUD populations, with several randomized controlled trials and systematic reviews highlighting its role in reducing drinking and increasing abstinence (53). Peer support programs, including AA and 12-step facilitation programs, are associated with improved rates of continuous abstinence at 12 months and are recommended as adjuncts to formal therapy (31). Regarding liver-related outcomes and survival, psychotherapy for AUD is associated with a reduced risk of incident ALD and hepatic decompensation (54). In a large cohort study, psychotherapy was linked to a lower rate of ALD development and reduced hepatic decompensation among patients with cirrhosis (54).

## MONITORING FOR ALCOHOL USE IN PATIENTS WITH LIVER DISEASE

Patients with AUD should be longitudinally monitored for ongoing alcohol use. Of the several available tools to monitor and quantify ongoing alcohol use, timeline follow-back (TLFB) is the most used tool. A calendar-based measure of dairy alcohol use over the previous weeks to a maximum period of up to 3 months has undergone extensive evaluation across diverse populations. TLFB is the most psychometrically adept self-reporting measure of drinking and is recommended by the FDA for use in clinical practice and research trials. Patients are asked to fill out the amount of alcohol used each day of the calendar within the specified time interval to assist with recall. Patients are encouraged to use diaries or appointment books and reflect on the alcohol used during different times of the week (55). Other ways to improve efficiency and accuracy include online options and staff support for data collection. As accuracy does not decrease over time, the tool is well-suited for studies that follow patients for long periods. A daily alcohol diary is another option and is an accurate tool with daily self-reported information by the patient. In addition to its use in short-term studies, the alcohol diary can complement TLFB data by avoiding long-term recall bias. Another advantage is that daily monitoring provides automated information on whether the use is above the threshold for alcohol-related harm, providing the patient with a regular motivation for change.

However, despite the best interviewing techniques by physicians, patients may not always be forthcoming regarding their alcohol use, and given the perceived stigma and fear of losing care, especially in the transplant waitlisted population, due to the fear of losing transplant candidacy. Biomarkers of alcohol metabolism can supplement the accuracy of Timeline Followback to detect ongoing alcohol use (Table 5).

**Table 5.** Biomarkers of alcohol use

No.	Biomarker	Window period	Sensitivity (%)	Specificity (%)	PPV (%)	NPV (%)
Direct biomarkers						
1	Phosphatidylethanol (Peth) (56)	30 d	100	96	85	100
2	Fatty acid ethyl ester (57,58)	2–4 d	100	90	4	23
3	Ethyl glucouronide (59)	48 h	71	98	90	95
Indirect biomarkers						
4	GGT (60)	2–6 wk	30–50	40–90	—	—
5	AST:ALT ratio	2–3 wk	Limited diagnostic accuracy			
6	Carbohydrate-deficient transferrin (61)	3 wk	44	99	96	71

ALT, alanine aminotransferase; AST, aspartate aminotransferase; GGT, gamma-glutamyltransferase; NPV, negative predictive value; PPV, positive predictive value.

### Direct biomarkers of alcohol use

Blood alcohol concentration has been used for years, especially in the inpatient and emergency department setting.

#### Phosphatidylethanolamine.

Can be used with a window period of detecting alcohol use for up to 3 to 4 weeks since last alcohol use due to its elimination half-life of 4 to 10 days (62). With a sensitivity of 94%–00% and specificity of 100%, the values (ng/mL) decrease overtime and correlate with degree of use (minimal or no use: <20, moderate use in the metabolic dysfunction and alcohol-related liver disease range: 20–200, and heavy use in ALD range: >200) (63–66). PEth values decrease over time and can be detected in approximately 64.3% of cases after 28 days of sobriety, which allows for longitudinal follow-up of alcohol use patterns/history in comparison with other biomarkers (67). As phosphatidylethanolamine (PeTH) resides in the red blood cell membrane, whole-blood samples are needed for testing, and caution must be exercised to avoid false positive values in the setting of hemolytic disease or postblood transfusion (68). As PeTH testing may have implications on the patient-provider relationship, clinicians should ideally discuss the rationale for testing with patients in advance and frame it as a routine component of care. If results are discordant with the patient's report of abstinence, a nonjudgmental and supportive discussion that invites the patient's perspective may help preserve trust while clarifying alcohol exposure.

#### Fatty acid ethyl ester.

Nonoxidative metabolites of ethanol, fatty acid ethyl ester (FAEE), have a detection window of only 4–5 days after alcohol consumption. Although quantification of FAEE levels does not correlate with the amount of alcohol use, it has been postulated to detect excessive drinking patterns. Compared with blood measurements, scalp hair FAEE measurements are thought to be more precise for detecting alcohol exposure.

#### Ethyl glucuronide.

Like FAEE, ethyl glucuronide (EtG) can be detected in blood and urine for up to 4–5 days after last alcohol use. It can also be detected in other body fluids, hair, and body tissues (69). Studies have shown hair EtG measurements to have sensitivity and specificity of 70%–90% for detecting alcohol use (70). However, EtG levels might be influenced by incidental exposure to alcohol (eg, hand sanitizers and mouthwash), and EtG in hair is also prone to esthetic therapies (71). Furthermore, its levels do not correlate linearly with alcohol consumption, as such, its utility remains limited to diagnostic rather than serial monitoring (72).

### Indirect biomarkers of alcohol use

#### Gamma-glutamyltransferase.

An early indicator of liver disease, elevated gamma-glutamyltransferase, is often found in patients with excessive alcohol use and ALD as a liver disease etiology (73). However, it has a low sensitivity of 30%–50% because elevated levels are noted in pancreatitis and in prostate disease.

#### AST and ALT.

Markers of hepatic inflammation and liver injury, AST and ALT values have poor diagnostic accuracy, especially at extremes of age (<30 or >70 years). Furthermore, AST is also located in the heart, muscle, kidney, and brain.

#### Carbohydrate-deficient transferrin.

It is a highly sensitive biomarker for heavy alcohol consumption, about 40 gm/d (3–4 standard drinks/d). Carbohydrate-deficient transferrin can be found for up to 3 weeks postalcohol consumption, and higher levels are noted in patients with chronic alcohol use (74). It is not influenced by comorbid conditions such as hypertension, asthma, depression, and gastrointestinal diseases and is not affected by prescription medication usage. However, conditions such as pregnancy, liver disease, and anemia do affect its levels, and the utility of carbohydrate-deficient transferrin in this patient population is limited (75,76).

The combination of Timeline Followback with biomarkers not only helps determine improvement in AUD but also helps define success. Although abstinence is the Food Drug Administration (FDA)-approved endpoint for success in clinical trials, reduction of alcohol use by 2 or more levels as defined by WHO is gaining momentum as an FDA-approved endpoint for success (77,78). These levels (gm/d) in men are defined as 0, 1–40, 41–60, 61–100, and >100 abstinence, low, moderate, high, and very high, respectively. The corresponding values in women are 0, 1–20, 21–40, 41–60, and >60. Once the endpoint is reached, maintaining the outcome for 1 year or more in addiction medicine can define a patient to be in remission from AUD, especially if the patient is abstaining or drinking below a moderate level of alcohol use (79). In patients with liver disease, alcohol use, even in the low range, is known to be associated with worse long-term outcomes (11,80–82). Hence, pending documentation of an acceptable cutoff on safe alcohol use in prospective validated multicenter clinical trials, abstinence is currently promoted as the successful endpoint in patients with ALD for alcohol use (77,78).

Timeline follow back (TLFB) is the most used tool to monitor ongoing alcohol use in clinical care and research trials.

Blood phosphatidylethanol (PeTH) measurement complements self-reported information using TLFB or AUDIT-C in accurately capturing ongoing alcohol use.

## SUMMARY AND FUTURE PROSPECTS

ALD is the most common cause of liver disease and cirrhosis-related morbidity, mortality, and healthcare burden. AUD, the most important factor associated with long-term outcomes, is rarely treated in the real world because of several patient and clinician-level barriers. Strategies are needed to (i) increase awareness and feasibility of provider screening for alcohol use at healthcare encounters, (ii) train gastroenterologists and hepatologists in addressing and managing AUD, especially on MAUD, and (iii) implement multidisciplinary care models integrating hepatologists, social work, counselors, and psychiatrists for managing ALD patients with moderate to severe AUD. Strategies are also needed to leverage digital and telehealth tools to manage AUD in patients with ALD in resource-limited or rural settings where specialist care is limited or unavailable (83). Furthermore, there remains an unmet clinical need of well-designed randomized controlled trials to develop safe and effective pharmacological interventions for AUD in patients with ALD.

GI and hepatology fellowship training programs should consider incorporating a 6–8-week curriculum targeting training on AUD to produce future generation of gastroenterologists and hepatologists who are comfortable in addressing and managing AUD in patients with ALD.

## CONFLICTS OF INTEREST

**Guarantor of the article:** Ashwani K. Singal, MD, MS, FACG.

**Specific author contributions:** Concept and design: A.K.S. Experiments and procedures: A.K.S. Data interpretation: S.P., N.T., and A.K.S. Writing the article draft: S.P. and N.T. All the authors reviewed, provided intellectual input, and approved the final draft.

**Financial support:** AKS is funded by NIAAA grant U01 AA026980-06, NIAAA grant P50AA024337-8301, and DHHS grant PON27462400004956.

**Potential competing interests:** ICMJE Statement: AKS serves as a consultant on the SBIR grant for Pleiogenic pharma; served as a DSMB member for phase-2b DUR-928 trial for Durect pharma; serves on advisory board for Orphalan, Disc Medicine, Ipsen, and Madrigal; speaks and writes for Medscape, CLD Foundation, Expert Perspectives, Gastro Endo News, Dynamed, Medical Education Speakers Network, Up-to-Date, Madrigal Pharma, and Practice Point. Other authors have no financial or other conflicts of interest to disclose.

## ABBREVIATIONS:

AH: alcohol-associated hepatitis
ALD: alcohol-associated liver disease
ALT: alanine aminotransferase
AST: aspartate aminotransferase
AUD: alcohol use disorder
BI: brief intervention
EtG: ethyl glucuronide
LSM: liver stiffness measurement
MAUD: medications for AUD
PeTH: phosphatidylethanol
TLFB: timeline follow back
